# Analyses of the Distribution Patterns of *Burkholderia pseudomallei* and Associated Phages in Soil Samples in Thailand Suggest That Phage Presence Reduces the Frequency of Bacterial Isolation

**DOI:** 10.1371/journal.pntd.0005005

**Published:** 2016-09-26

**Authors:** Patoo Withatanung, Narisara Chantratita, Veerachat Muangsombut, Natnaree Saiprom, Ganjana Lertmemongkolchai, Jochen Klumpp, Martha R. J. Clokie, Edouard E. Galyov, Sunee Korbsrisate

**Affiliations:** 1 Department of Immunology, Faculty of Medicine Siriraj Hospital, Mahidol University, Bangkok, Thailand; 2 Department of Microbiology and Immunology, Faculty of Tropical Medicine, Mahidol University, Bangkok, Thailand; 3 Mahidol-Oxford Tropical Medicine Research Unit, Faculty of Tropical Medicine, Mahidol University, Bangkok, Thailand; 4 Department of Clinical Immunology, Faculty of Associated Medical Sciences, Khon Kaen University, Khon Kaen, Thailand; 5 Institute of Food, Nutrition and Health, ETH Zurich, Zurich, Switzerland; 6 Department of Infection, Immunity and Inflammation, University of Leicester, Leicester, United Kingdom; Institut Pasteur, FRANCE

## Abstract

**Background:**

*Burkholderia pseudomallei* is a soil saprophytic bacterium that causes melioidosis. The infection occurs through cutaneous inoculation, inhalation or ingestion. Bacteriophages (phages) in the same ecosystem may significantly impact the biology of this bacterium in the environment, and in their culturability in the laboratory.

**Methods/Principal Findings:**

The soil samples were analysed for the presence of bacteria using culture methods, and for phages using plaque assays on *B*. *pseudomallei* strain 1106a lawns. Of the 86 soil samples collected from northeastern Thailand, *B*. *pseudomallei* was cultured from 23 (26.7%) samples; no phage capable of infecting *B*. *pseudomallei* was detected in these samples. In contrast, phages capable of infecting *B*. *pseudomallei*, but no bacteria, were present in 10 (11.6%) samples. *B*. *pseudomallei* and their phages were co-isolated from only 3 (3.5%) of soil samples. Since phage capable of infecting *B*. *pseudomallei* could not have appeared in the samples without the prior presence of bacteria, or exposure to bacteria nearby, our data suggest that all phage-positive/bacteria-negative samples have had *B*. *pseudomallei* in or in a close proximity to them. Taken together, these findings indicate that the presence of phages may influence the success of *B*. *pseudomallei* isolation. Transmission electron microscopy revealed that the isolated phages are podoviruses. The temperate phages residing in soil-isolated strains of *B*. *pseudomallei* that were resistant to the dominant soil borne phages could be induced by mitomycin C. These induced-temperate phages were closely related, but not identical, to the more dominant soil-isolated phage type.

**Conclusion/Significance:**

The presence of podoviruses capable of infecting *B*. *pseudomallei* may affect the success of the pathogen isolation from the soil. The currently used culture-based methods of *B*. *pseudomallei* isolation appear to under-estimate the bacterial abundance. The detection of phage capable of infecting *B*. *pseudomallei* from environmental samples could be a useful preliminary test to indicate the likely presence of *B*. *pseudomallei* in environmental samples.

## Author Summary

*Burkholderia pseudomallei* is a motile, Gram-negative bacterium that causes melioidosis. The disease is endemic in Southeast Asia and Northern Australia and can be fatal. In the zones of endemicity, *B*. *pseudomallei* are commonly found in soils, and the bacteria can move to the surface during the rainy season. In Thailand, rice farmers rarely wear protective footwear, and thus they are exposed to a risk of infection with *B*. *pseudomallei* by cutaneous inoculation. Biological factors such as bacteriophages (phages), viruses of bacteria, present in the same ecosystem as *B*. *pseudomallei* may affect the population dynamics of this bacterium in the environment. In order to study this, we have investigated the distribution patterns of *B*. *pseudomallei* and associated phages in nature and the impact of these phages on the bacterial culturability. This is the first study demonstrating that the presence of phages capable of infecting *B*. *pseudomallei* may affect the success of the pathogen isolation from the soil. Currently culture-based methods of *B*. *pseudomallei* appear to under-estimate the bacterial abundance.

## Introduction

*Burkholderia pseudomallei* is a motile, Gram-negative, non-spore-forming bacterium that causes melioidosis [1]. The disease is endemic in Southeast Asia and Northern Australia. The clinical manifestations of melioidosis range from localized infection to sepsis and death, with pneumonia being the most common presentation [2]. Treatment with ineffective antimicrobials may result in case fatality rate exceeding 70% [3]. Currently, there is no licensed vaccine available. The bacteria are intrinsically resistant to many antibiotics. In a typical clinical case, parenteral treatment with ceftazidime is given for at least 10 days, followed by oral treatment with a four-drug combination (chloramphenicol, doxycycline, trimetoprim-sulfamethoxazole) for 20 weeks. Due to its aerosol infectivity, the high mortality rate and the absence of effective human vaccine available for the treatment of melioidosis [1, 4], *B*. *pseudomallei* has been recognised as a potential bio-threat agent, and it has been listed as category B disease/agents by the U.S. Centers for Disease Control and Prevention [1, 5].

In the zones of endemicity, *B*. *pseudomallei* are commonly found in clay soils at a depth of about 25–45 cm, and the bacteria can move to the surface during the rainy season [6]. In Thailand, rice farmers rarely wear protective footwear, and thus they are exposed to a risk of infection with *B*. *pseudomallei* by cutaneous inoculation. Moreover, heavy rain may create aerosols contaminated with the bacteria, and this can result in the inhalation of the organism [7]. The existence of these risk factors is correlated with a high incidence of melioidosis in the rainy seasons. There are reports that temperature, pH, and water content in the soil might affect *B*. *pseudomallei* survival [8, 9]. In addition to soil physicochemical properties, biological factors such as bacteriophages (phages) present in the same ecosystem as *B*. *pseudomallei* may affect the density of this bacterium in the environment. Our previous work has revealed that *B*. *pseudomallei* phages can be readily isolated from the soil environment [10, 11].

Phages are the most abundant life form on Earth, and they are frequently found to co-exist with their bacterial hosts with approximately 10 phage particles for every bacterial cell. They can be found in most environments, such as sewage, soil, and water [12, 13]. In general phages either follow a lytic or a lysogenic life cycle so they can shape microbial communities by lysing their hosts or alternatively by lysogenizing them where they may provide phenotypic advantages to recipient bacteria [14, 15]. Phages are thus considered to have major role with respect to bacterial abundance, population structure and diversity in a variety of environments including soil.

There is only limited information available about the role of temperate phages in the virulence of *B*. *pseudomallei*. The *B*. *pseudomallei* K96243 genome is organized into two circular chromosomes [16]. The genome is highly mobile with sixteen genomic islands constituting over 10% of the total genome. Eight of these islands have some prophage like characteristics, and one genomic island was shown to encode phiK96243 phage [16, 17]. More recently, Gatedee et al [10] reported the isolation of ΦBp-AMP1 and other *B*. *pseudomallei* phages from soil samples. Almost all of the isolated *B*. *pseudomallei* phages were podoviruses, suggesting that such phages are dominant in the environment. Interestingly, there are no ΦBp-AMP-1-like prophages detected in the sequenced *B*. *pseudomallei* strains. This lack of such prophages may due to the commonly used culture temperature of 40°C for *B*. *pseudomallei* isolation, which results in the phage induction, and thus remaining bacteria are phage-free. Further characterization by our group [11] showed that a class of temperate podoviruses that target *B*. *pseudomallei* go through a lytic cycle at 37°C, whereas at 25°C they infect their host bacteria but remain temperate. These lysogens are relatively stable at lower temperature but when the bacteria are incubated at 37°C they are induced with a high frequency. Similarly, when lysogens are passaged through mice, in most cases the prophages are induced and thus the bacteria are killed. However, the small numbers of bacteria that are recovered are phage-free, suggesting that on entry to the mouse these bacteria have lost their phages without being killed.

In order to assess whether *B*. *pseudomallei* could be co-isolated with their dominant phages from the environment, multiple soil samples were collected from northeastern Thailand where melioidosis is endemic. The presence of *B*. *pseudomallei* in the samples was assessed by culture. Phages were detected using spot assays and their presence confirmed by plaque assays on *B*. *pseudomallei* strain 1106a lawns. In the majority of samples that contained *B*. *pseudomallei* or phages, only one of the partners was present; bacteria and phages were co-isolated only in a small number of samples. The *B*. *pseudomallei* strains that were co-isolated with bacteria were found to be relatively stable lysogens and were resistant to infection from the dominant soil phage type. Furthermore their own resident temperate phages could be induced from the bacteria using mitomycin C (MMC). The induced phages were compared to the soil-isolated phages by assessing their morphology using Transmission Electron Microscopy (TEM) and restriction analysis of the phage DNA.

## Materials and Methods

### Bacterial strains and culture conditions

*Burkholderia pseudomallei* strain 1106a was chosen as a propagation strain for phage isolation and purification because its genome lacks prophage islands [18], and because it has a multilocus sequence type (ST) 70, which is the most abundant genotype in northeastern Thailand [19]. All *B*. *pseudomallei* strains were cultured on Luria-Bertani (LB) agar (Hardy Diagnostics, USA) and incubated at 37°C for 18–24 hours. To obtain mid-log phase cells, 10 μl of overnight-cultured *B*. *pseudomallei* were sub-cultured into 3 ml of LB broth (Thermo Fisher Scientific, USA) and incubated at 37°C for approximately 4–6 hours.

### Soil sample collection for *B*. *pseudomallei* and phages isolations

One hundred and one soil samples were collected from a backyard and a rice paddy field of a melioidosis patient in the rainy season from Roi-Et province, northeast Thailand, which is an endemic area of melioidosis disease. The soil in this area is sandy loam soil. Sharp-ended polyvinyl chloride tubes were used to collect soil from a depth of 30 cm and 2.5x2.5 meters apart according to Wuthiekanun et al [20].

For *B*. *pseudomallei* isolation, 10 g of soil samples were weighed and added directly into 10 ml of threonine-basal salt solution (TBSS) plus Colistin 50 μg/ml (TBSS-C50), mixed, and then incubated at 40°C for 48 hours. Ten microliters of the broth culture was subcultured onto Ashdown agar plates and incubated at 40°C in air for 4 days as described previously [21]. Putative *B*. *pseudomallei* showing typical colonies on Ashdown’s agar were confirmed by the latex agglutination test [22] and arabinose assimilation test [23]. *B*. *pseudomallei* were characterized as arabinose non-assimilators and latex agglutination test positive.

Isolation of phages from soil was performed according to previously described [10]. Essentially, 2 g of soil were transferred into 10 ml LB broth plus 5 mM CaCl_2_ before mixing thoroughly by inversion and incubated at least 1 hour at room temperature. Then, the mixture was centrifuged at 4000xg for 20 minutes and supernatant was collected for filtration through a 0.22-μm filter membrane. This phage filtrate preparation was used for phages isolation by spot assay [10] and confirmed for the presence of phages by double agar plaque assay [24] using *B*. *pseudomallei* strain 1106a as bacterial host.

### Isolation of mitomycin C-induced temperate phages from *B*. *pseudomallei*

Temperate phages were induced from *B*. *pseudomallei* according to Wright et al [25]. In brief, freshly prepared MMC (Sigma-Aldrich, USA) was added to 10 ml of mid-log phase *B*. *pseudomallei* culture to a final concentration of 250 ng/ml. The optical density (OD_600nm_) was measured every 2–4 hours over 24 hours. After 24 hours of incubation, the sample was centrifuged at 4000×*g* for 10 minutes and the supernatant containing phages was passed through a 0.45-μm filter membrane (Whatman, UK). The presence of phages was confirmed using the double agar plaque assay [24].

### Transmission Electron Microscopy

Negative staining was performed for phage morphology examination by TEM (JEOL 1230, Japan). Phages (approximately 10^6^−10^8^ particles/ml) were fixed with 2.5% glutaraldehyde in 0.1 M phosphate-buffered saline (PBS; pH 7.3) for 3 hours, and a drop of this solution was dropped onto a carbon-coated copper grid (200 mesh). The excess liquid was removed by filter paper and the grid was dried. A drop of 2% phosphotungstate was added for 1 minute, and the excess liquid was removed with a piece of filter paper before further drying. Digital images were captured using a GATAN Orius 1k camera with associated analysis software.

### Phage DNA extraction and restriction enzyme analysis

Phages were concentrated using polyethylene glycol (PEG; Sigma-Aldrich, USA) precipitation as previously described [26]. After mixing with PEG8000, the phage suspension was centrifuged at 11,000×*g* for 25 minutes at 4°C and the pellet was re-suspended in SM buffer (Storage Media; 100 mM NaCl, 10 mM MgSO_4_.7H_2_O, 50 mM Tris-HCl, pH7.5). DNaseI (14 mg/ml) and RNaseA (30 mg/ml) (Thermo Fisher Scientific, USA) were then added, and the mixture was incubated at 37°C for 1 hour to eliminate bacterial nucleic acids. Following phenol/chloroform extraction, the aqueous phase was precipitated using absolute ethanol. The precipitated phage DNA was washed, dissolved in sterile deionized water, and stored at -20°C until use.

For restriction enzyme analysis, the isolated phage DNA was digested with *Bst*BI or *Mlu*I restriction endonucleases using the manufacturer’s recommended conditions. Digested DNA was separated by agarose gel electrophoresis, and images were captured using GeneSnap acquisition software (Syngene, UK).

### Polymerase chain reaction (PCR) and DNA sequencing

PCR was performed using primers specific for the phage tail tubular protein B gene. The forward (5′-TAAGGTAACAGGCAGCTACG-3′) and reverse (5′-ATTGAGCACGAAGCA GAACG-3′) primers were designed from *B*. *pseudomallei* ΦBp-AMP1 [10]. The reaction mixture contained 10 ng of phage DNA, 0.2 units of *Taq* DNA polymerase (Bioline, London, UK), 0.4 μM of each primer, 250 μM of each deoxynucleotidetriphosphate (dNTP), 1×PCR buffer, and 2.0 mM MgSO_4_. Thermal cycling was performed in a TProfessional thermocycler machine (Biometra, Germany) with the following parameters: 95°C for 2 minutes, followed by 35 cycles of 94°C for 45 seconds, 51°C for 45 seconds, and 72°C for 1 minute, and a final extension at 72°C for 10 minutes. The amplified products were analyzed by agarose gel electrophoresis.

## Results and Discussion

### *B*. *pseudomallei* and their phages in soil collected from northeastern Thailand

**Fig 1:**
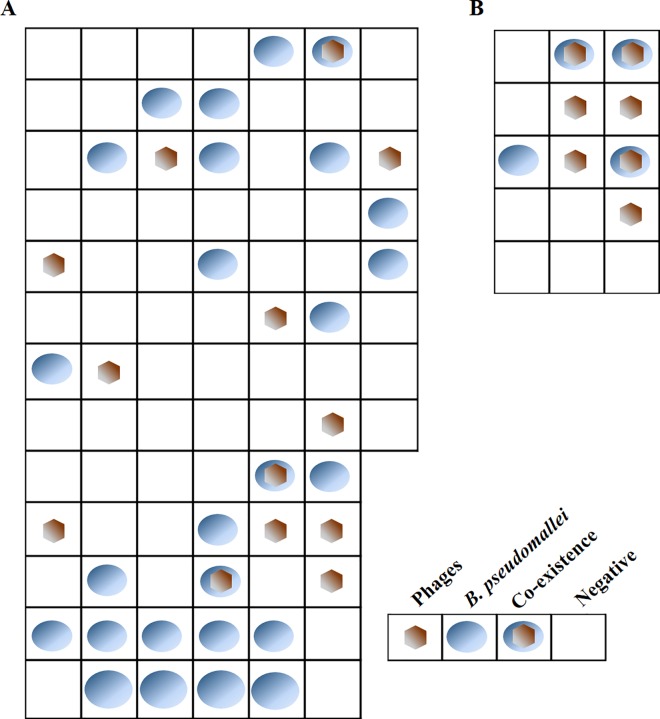
Distributions of *B*. *pseudomallei* and their phages in soil samples. The 86 (A) and 15 (B) soil samplings were collected from Roi-Et province in northeastern Thailand during rainy season. Each square represents a 2.5 m x 2.5 m area of the field, in which soil sample was taken at a depth of 30 cm. The presence of *B*. *pseudomallei* and phages was assessed in each sample and the results are shown in this Figure.

Northeastern Thailand is the endemic area of melioidosis where humans can be infected by having contact with *B*. *pseudomallei* residing in soil or water. Bacterial density in the environment is a crucial risk factor for infection. Despite a relatively high abundance of *B*. *pseudomallei* phages, little is known about their distribution and their impact on the *B*. *pseudomallei* population in natural habitats. To initiate research in this area, we collected 86 soil samples from a backyard of a melioidosis patient in the Roi-Et province, and processed the samples for the isolation of *B*. *pseudomallei* and their phages. Neither *B*. *pseudomallei*, nor phage capable of infecting *B*. *pseudomallei* could be detected in 50 samples. This could be due to the absence of bacteria and phages in these samples, or that their presence was at levels below the detection limit for the methods used in this study. Twenty-three (26.7%) soil samples were positive for *B*. *pseudomallei* but not phages, as assessed by the growth of bacterial colonies on Ashdown agar plates (Fig 1A).

The isolation of *B*. *pseudomallei* from soil was carried out in accordance with recommendations provided by an authoritative paper in the field [27]. The authors of this paper clearly state that the incubation temperature of 40°C was suggested based on evidence that it allows good growth of *B*. *pseudomallei* but inhibits many other soil bacteria.

In our project, we have also tried to incubate soil samples in liquid broth at 25°C (instead of 40°C) before plating at 25°C and found that many other bacteria over growth on the plate, even though there were antibiotics in the culture medium. *B*. *pseudomallei* colonies growing at 25°C on agar plate are hard to find due to the very small (pin-point) colony size and overgrowth of other bacterial species. In addition, it is hard to identify the suspected *B*. *pseudomallei* as colonies are not suitable for further confirmation by latex agglutination test due to the size and many contaminating colonies.

To isolate phages, spot tests were used to test extracts from these soil samples, which were spotted on the *B*. *pseudomallei* 1106 a lawns. Ten (11.6%) of the soil samples were positive for only the phages, as assessed by phage plaques formation on the lawns of *B*. *pseudomallei* strain 1106a (Fig 1A). Note that we did not enrich for the presence of phages and thus would not have been able to observe phage presence if they were present at less than 5 plaque forming units (PFU)/ml of suspension which corresponds to 25 PFU/g of soil (This is based on the detection method where 2 g of soil is suspended in 10 ml of LB broth, and then 200 μl aliquot is taken to quantify plaques by the double agar plaque assay). No *B*. *pseudomallei* colonies were grown on the Ashdown agar plates from these phage positive soil samples, when they were processed and plated. *B*. *pseudomallei* and their phages were only detected in 3 (3.5%) of the samples analysed. Since phages capable of infecting *B*. *pseudomallei* could not have appeared in the samples without the prior presence of bacteria, or exposure to bacteria nearby, our data suggest that all phage-positive/bacteria-negative samples have had *B*. *pseudomallei* (or possibly *B*. *thailandensis*) in or in a close proximity to them, but these bacteria were killed by the phages either before or during the sampling itself or processing the samples. Thus, it could be concluded that currently adopted culture-based methods of *B*. *pseudomallei* detection in the environmental samples appear to under-estimate the bacterial abundance, at least in our sampling area. This under-estimation is likely to be at least due to the action of the phages e.g. the commonly used culture temperature of 40°C for *B*. *pseudomallei* isolation may lead to prophage induction and the bacteria are killed [11]. Since these phages appear to be abundant in the environment alongside with the bacteria, we propose that phage plaque assay on an indicator *B*. *pseudomallei* host could be a useful preliminary test to indicate the likely presence of *B*. *pseudomallei* in environmental samples. Although the results of such a test should be treated with some caution because many phages that infect *B*. *pseudomallei* can also infect the closely related *B*. *thailandensis*, they can provide a simple and rapid assessment of the likely presence of *B*. *pseudomallei* in the environment.

### Phages that are present as *B*. *pseudomallei* lysogens that coexist with free phages in the soil could be induced using MMC

To assess if the pattern of differential *B*. *pseudomallei*-phage detection in the soil samples was common, we repeated the experiment but sampled a smaller number of samples (n = 15) in a rice field three kilometers from the first sampling site. A similar pattern of bacteria and phage distribution was observed: one sample was positive for only *B*. *pseudomallei*, four samples positive for only the phage, and three samples positive for both the bacteria and the phage (Fig 1B).

**Fig 2:**
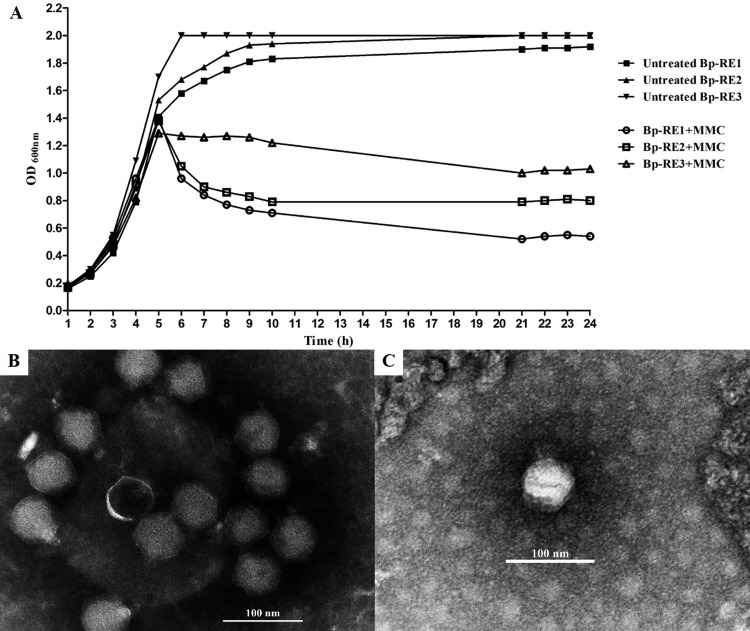
The growth of mitomycin C (MMC)-treated *B*. *pseudomallei* cultures and Transmission Electron Microscopy assessment of the induced phages. Growth of different strains following MMC induction. The MMC-treated cultures show a decline in optical density (OD_600nm_) after addition of MMC that is not observed in the untreated culture (A). Soil-isolated (B) and MMC-induced temperate (C) phages have similar icosahedral heads with short, non-contractile tails, which are characteristic of phages in the *Podoviridae* family.

All three of the freshly soil-isolated *B*. *pseudomallei* (RE1, RE2 and RE3) which appeared to coexist with phages in the soil were chosen for phage induction by MMC treatment. The bacteria were subcultured at least 3–4 times to avoid phage contamination before culturing in LB broth with or without MMC treatment. Growth curves of MMC-treated *B*. *pseudomallei* isolates RE1-3 indicated the induction of temperate phages following treatment (Fig 2A). The optical density (OD_600nm_) of MMC-treated *B*. *pseudomallei* strains was significantly reduced compared to the OD values of untreated bacteria. To confirm successful prophage induction, the supernatant of the MMC-treated *B*. *pseudomallei* cultures were subjected to a plaque assay. The results showed that the induced phages could infect a test strain of *B*. *pseudomallei* 1106a and yielded clear plaques approximately 3–5 mm in diameter; no plaque formation was detected when using *B*. *pseudomallei* RE1, RE2 or RE3 as the bacterial host. The likely explanation for the apparent resistance of these strains is the presence of prophage within these host strains.

We observed no difference in plaque morphology on the *B*. *pseudomallei* 1106a lawns for the MMC-induced phages and no difference in plaque morphology between the MMC-induced phages and our previously isolated free phages ΦBp-AMP1 [10] (S1 Fig). The temperate phages derived from *B*. *pseudomallei* strains RE1-3 were subsequently designated ΦBp-RE1, ΦBp-RE2, and ΦBp-RE3, respectively. No phage plaques were observed when samples from the control cultures incubated without MMC were spotted on the *B*. *pseudomallei* 1106a lawns, which indicates that there is no spontaneous release of the phages from the cultures of RE1-3 strains (Fig 2A). However, it is possible that the induced phage (if any) is not able to infect *B*. *pseudomallei* 1106a indicator bacteria or the number of induced phages is lower the detection limit (5 PFU/ml). Together, these data suggest a successful temperate phage induction from all three soil-phage-resistant *B*. *pseudomallei* and that the induced temperate phages could infect *B*. *pseudomallei* strain 1106a.

The temperate phages ΦBp-RE1, ΦBp-RE2, and ΦBp-RE3 were plaque-purified at least five times, and the phage morphology was assessed by TEM. All of the phages had icosahedral head (diameter ~ 58 nm) with a short and non-contractile tail, which is characteristic of phages in the *Podoviridae* family (Fig 2B and 2C). The genome sizes of these phages, as assessed by pulsed-field gel electrophoresis, were estimated at approximately 45 Kb (S2 Fig). This is similar with that for the ΦBp-AMP1 phages characterized in our previous work [10]. In addition, these data provide a likely explanation to why these three isolates were originally found co-isolated with the free soil phages: it is likely that they are lysogenized with the phages identical or at least highly similar to the dominant podovirus type abundantly found in soil, and this lysogeny provides a protection from the attack by the same or a related phage.

### MMC-induced and soil-isolated *B*. *pseudomallei* phages are highly similar but not identical

**Fig 3:**
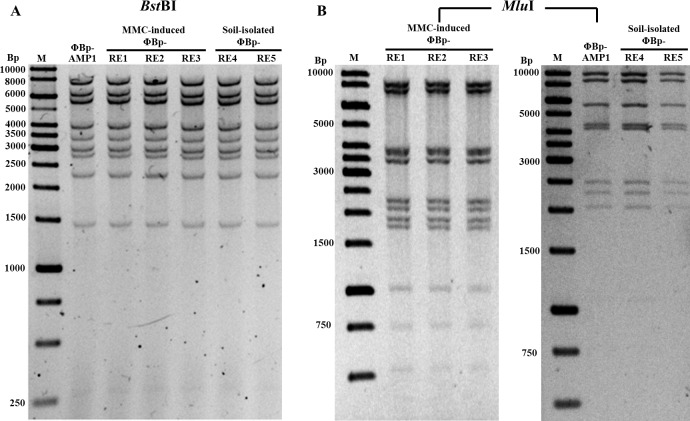
Restriction enzyme analyses of *B*. *pseudomallei* phages. Genomic DNA extracted from soil isolated (ΦBp-RE4-5) and MMC-induced (ΦBp-RE1-3) *B*. *pseudomallei* phages were digested with restriction enzyme *Bst*BI (A) or *Mlu*I (B) and analyzed using agarose gel electrophoresis. Different DNA patterns were observed when digested with the *Mlu*I restriction enzyme. A 1-kb DNA ladder was included as a DNA marker.

To further compare the genetic features between MMC-induced *B*. *pseudomallei* phages (ΦBp-RE1-3) and two newly isolated free phages (ΦBp-RE4-5), *Bst*BI and *Mlu*I restriction enzyme digestion analyses of the phage genomic DNA were carried out. No significant difference between the *Bst*BI restriction enzyme digestion patterns among the three MMC-induced phages and free phages were observed and these phages have the same calculated genome size of approximately 45 Kb (Fig 3A). However, different DNA patterns were observed when the *Mlu*I restriction digestion profiles were analyzed (Fig 3B). It appears that additional *Mlu*I recognition sites are present in the genomes of the induced phages, resulting in the alterations of the *Mlu*I digestion profile of the induced phages compared to that of the dominant soil-isolated ones.

**Fig 4:**
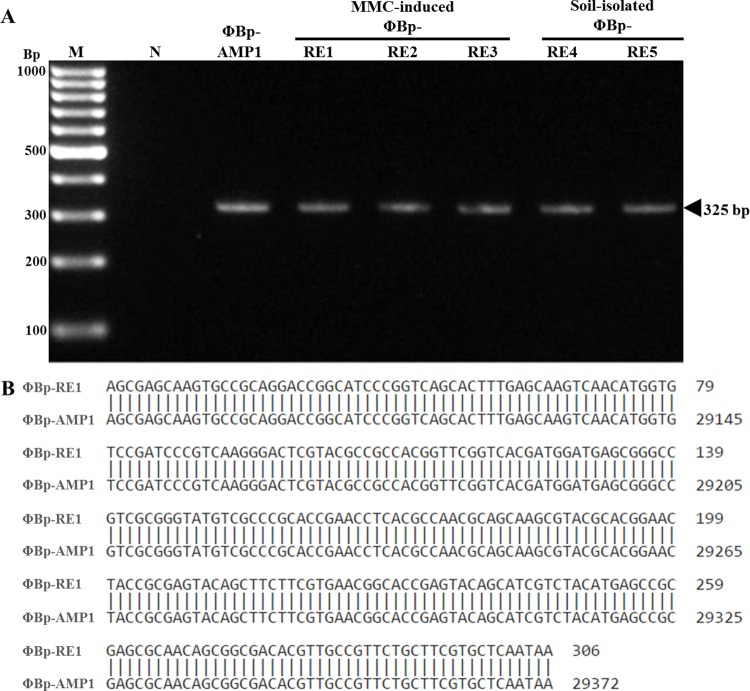
PCR amplification and DNA sequence analysis of the DNA fragment encoding the *B*. *pseudomallei* phage tail tubular protein B. DNA from soil-isolated and MMC-induced temperate phages were used as a template to PCR amplify the fragment of a gene encoding the phage tail tubular protein B. The 325-bp DNA fragment was detected in each case. A negative PCR control (N) and 100-bp DNA marker were included (A). Nucleotide sequences comparison of the phage tail tubular protein B DNA amplified from ΦBp-RE1 and ΦBp-AMP1 is shown (B).

In addition to the restriction enzyme digestion analyses, PCR analysis with primers specific to a gene encoding the tail tubular protein B of ΦBp-AMP1 [10] were carried out on the MMC-induced temperate and the free phages. An approximately 325-bp-long DNA fragment was amplified when each of the phage samples was used as a template (Fig 4A). To explore whether they have the same nucleotide sequences, DNA sequencing of the amplified DNA fragment was carried out. The amplified DNA fragment from MMC-induced temperate phages (ΦBp-RE1-3) and free phages (ΦBp-RE4-5) showed identical nucleotide sequences and had 100% nucleotide sequences identity to ΦBp-AMP1 (Fig 4B), further suggesting the close similarity between these phages.

The *B*. *pseudomallei* temperate podoviruses induced in this study differ from those observed in a previous study [26], in which MMC treatment of a clinical isolated *B*. *pseudomallei* induced a temperate virus (ΦP27) that belonged to the *Siphoviridae* family. In addition to MMC, Ronning et al [18] reported the induction of prophages from *B*. *pseudomallei* strains Pasteur 52237, E12, 644 by UV light, and found that the released viruses were from the *Siphoviridae* and *Myoviridae* families. Taken together, these data indicate the abundance of temperate phages in the environmental *B*. *pseudomallei* strains. The impact of such phages on *B*. *pseudomallei* biology is largely unknown. However, we have previously suggested that a group of temperature-dependent podoviruses are likely to significantly influence many aspects of *B*. *pseudomallei* existence in the environment. They are also likely to impact our ability to detect, and correctly enumerate bacteria from environmental samples [11]. The research presented here is consistent with our previous data and provides further evidence for the importance of this phage group.

### Conclusion

This is the first study that has assessed the presence of both *B*. *pseudomallei* and their phages in the same soil samples collected from the endemic area of melioidosis. The podovirus capable of infecting *B*. *pseudomallei* appear to be abundantly present in the soil. Currently adopted culture-based methods used for the detection of *B*. *pseudomallei* in the environmental samples appear to under-estimate the bacterial abundance due to the action of the phages. Some environmental isolates of *B*. *pseudomallei* appear to be relatively stably lysogenized by the podoviruses. Temperate phages could be induced from such *B*. *pseudomallei* strains isolated from soil by MMC. Restriction enzyme analyses indicated that MMC-induced phages are closely related to common free soil-isolated phages [10, 11]. Further investigation of the phage-host infection networks and dynamics of complex phage-host communities will help us to reveal the role of phages player in shaping the density in the soil of the life-threatening bacterial pathogen *B*. *pseudomallei*.

## Supporting Information

S1 Fig Plaque morphology of *B*. *pseudomallei* phages. Plaque morphology of soil-isolated phage ΦBp-AMP1 (A) and MMC-induced phage ΦBp-RE1 (B) on the *B*. *pseudomallei* 1106a lawns.
(TIF)

S2 Fig Pulsed-field gel electrophoresis of *B*. *pseudomallei* phage DNA. Genomic DNA extracted from MMC-induced phages ΦBp-RE1 (lane 1), ΦBp-RE2 (lane 2) and ΦBp-RE3 (lane 3) were subjected to pulsed-field gel electrophoresis. The genome sizes of these phages were estimated at approximately 45 Kb.
(TIF)

## References

[1] Cheng AC, Currie BJ. Melioidosis: epidemiology, pathophysiology, and management. Clin Microbio Rev. 2005; 18(2):383–416. 10.1128/CMR.18.2.383–416.2005 15831829 PMC1082802

[2] Chaowagul W, White NJ, Dance DA, Wattanagoon Y, Naigowit P, Davis TM, et al Melioidosis: a major cause of community-acquired septicemia in northeastern Thailand. J Infect Dis. 1989; 159(5):890–899. . 2708842 10.1093/infdis/159.5.890

[3] Limmathurotsakul D, Golding N, Dance DA, Messina JP, Pigott DM, Moyes CL, et al Predicted global distribution of *Burkholderia pseudomallei* and burden of melioidosis. Nat Microbiol. 2016; 1(1). pii: 15008 27571754 10.1038/nmicrobiol.2015.8

[4] Wiersinga WJ, van der Poll T, White NJ, Day NP, Peacock SJ. Melioidosis: insights into the pathogenicity of *Burkholderia pseudomallei*. Nat Rev Microbiol. 2006; 4(4):272–282. . 16541135 10.1038/nrmicro1385

[5] Rotz LD, Khan AS, Lillibridge SR, Ostroff SM, Hughes JM. Public health assessment of potential biological terrorism agents. Emerg Infect Dis. 2002; 8(2):225–230. . 11897082 10.3201/eid0802.010164 PMC2732458

[6] Currie BJ. *Burkholderia pseudomallei* and *Burkholderia mallei*: Melioidosis and Glanders. Oxford: Churchill Livingstone; 2004.

[7] Ip M, Osterberg LG, Chau PY, Raffin TA. Pulmonary melioidosis. Chest. 1995; 108(5):1420–1424. . 7587451 10.1378/chest.108.5.1420

[8] Tong S, Yang S, Lu Z, He W. Laboratory investigation of ecological factors influencing the environmental presence of *Burkholderia pseudomallei*. Microbiol and Immunol. 1996; 40(6):451–453. . 8839431 10.1111/j.1348-0421.1996.tb01092.x

[9] Chen YS, Chen SC, Kao CM, Chen YL. Effects of soil pH, temperature and water content on the growth of *Burkholderia pseudomallei*. Folia Microbiol. 2003; 48(2):253–256. . 12800512 10.1007/BF02930965

[10] Gatedee J, Kritsiriwuthinan K, Galyov EE, Shan J, Dubinina E, Intarak N, et al Isolation and characterization of a novel podovirus which infects *Burkholderia pseudomallei*. Virol J. 2011; 8:366 10.1186/1743-422X-8-366 21791081 PMC3169511

[11] Shan J, Korbsrisate S, Withatanung P, Adler NL, Clokie MR, Galyov EE. Temperature dependent bacteriophages of a tropical bacterial pathogen. Front Microbiol. 2014; 5:599 10.3389/fmicb.2014.00599 25452746 PMC4231975

[12] Clokie MR, Millard AD, Letarov AV, Heaphy S. Phages in nature. Bacteriophage. 2011; 1(1):31–45. 10.4161/bact.1.1.14942 21687533 PMC3109452

[13] Heldal M, Bratbak G. Production and decay of viruses in aquatic environments. Mar Ecol Prog Ser. 1991; 72:205–212.

[14] Fortier LC, Sekulovic O. Importance of prophages to evolution and virulence of bacterial pathogens. Virulence. 2013; 4(5):354–365. 10.4161/viru.24498 23611873 PMC3714127

[15] Feiner R, Argov T, Rabinovich L, Sigal N, Borovok I, Herskovits AA. A new perspective on lysogeny: prophages as active regulatory switches of bacteria. Nat Rev Microbiol. 2015; 13(10):641–650. 10.1038/nrmicro3527 . 26373372

[16] Holden MT, Titball RW, Peacock SJ, Cerdeno-Tarraga AM, Atkins T, Crossman LC, et al Genomic plasticity of the causative agent of melioidosis, *Burkholderia pseudomallei*. Proc Natl Acad Sci USA. 2004; 101(39):14240–14245. 10.1073/pnas.0403302101 15377794 PMC521101

[17] Summer EJ, Gill JJ, Upton C, Gonzalez CF, Young R. Role of phages in the pathogenesis of *Burkholderia*, or 'Where are the toxin genes in *Burkholderia* phages?'. Curr Opin Microbiol. 2007; 10(4):410–417. 10.1016/j.mib.2007.05.016 17719265 PMC2064068

[18] Ronning CM, Losada L, Brinkac L, Inman J, Ulrich RL, Schell M, et al Genetic and phenotypic diversity in *Burkholderia*: contributions by prophage and phage-like elements. BMC Microbiol. 2010; 10:202 10.1186/1471-2180-10-202 20667135 PMC2920897

[19] Vesaratchavest M, Tumapa S, Day NP, Wuthiekanun V, Chierakul W, Holden MT, et al Nonrandom distribution of *Burkholderia pseudomallei* clones in relation to geographical location and virulence. J Clin Microbiol. 2006; 44(7):2553–2557. 10.1128/JCM.00629-06 16825379 PMC1489466

[20] Wuthiekanun V, Dance DA, Wattanagoon Y, Supputtamongkol Y, Chaowagul W, White NJ. The use of selective media for the isolation of *Pseudomonas pseudomallei* in clinical practice. J Med Microbiol. 1990; 33(2):121–126. 10.1099/00222615-33-2-121 . 2231678

[21] Limmathurotsakul D, Wuthiekanun V, Amornchai P, Wongsuwan G, Day NP, Peacock SJ. Effectiveness of a simplified method for isolation of *Burkholderia pseudomallei* from soil. Appl Environ Microbiol. 2012; 78(3): 876–877. 10.1128/AEM.07039-11 22101048 PMC3264119

[22] Duval BD, Elrod MG, Gee JE, Chantratita N, Tandhavanant S, Limmathurotsakul D, et al Evaluation of a latex agglutination assay for the identification of *Burkholderia pseudomallei* and *Burkholderia mallei*. Am J Trop Med Hyg. 2014; 90(6):1043–1046. 10.4269/ajtmh.14-0025 24710616 PMC4047727

[23] Smith MD, Angus BJ, Wuthiekanun V, White NJ. Arabinose assimilation defines a nonvirulent biotype of *Burkholderia pseudomallei*. Infect Immun. 1997; 65(10):4319–43121. 9317042 10.1128/iai.65.10.4319-4321.1997 PMC175618

[24] Sambrook J, Russell DW. Molecular Cloning: A Laboratory Manual. 3rd ed. New York: Cold Spring Harbor Laboratory Press; 2001.

[25] Wright EE, Elliman JR, Owens L. Induction and characterization of lysogenic bacteriophages from *Streptococcus iniae*. J Appl Microbiol. 2013; 114(6):1616–1624. 10.1111/jam.12192 . 23490045

[26] Khanthawud K, Tattawasart U, Wongratanacheewin S, Manjai A. Isolation and charaterization of a lysogenic phage from *Burkholderia pseudomallei*. KKU Res J. 2011; 11(3):41–48.

[27] Limmathurotsakul D, Dance DA, Wuthiekanun V, Kaestli M, Mayo M, Warner J, et al Systematic review and consensus guidelines for environmental sampling of *Burkholderia pseudomallei*. PLoS Negl Trop Dis. 2013; 7(3):e2105 10.1371/journal.pntd.0002105 23556010 PMC3605150

