# Supplementary figures and images for: Analyses of the Distribution Patterns of Burkholderia pseudomallei and Associated Phages in Soil Samples in Thailand Suggest That Phage Presence Reduces the Frequency of Bacterial Isolation
Source: PLoS Negl Trop Dis. 2016 Sep 26;10(9):e0005005. doi: 10.1371/journal.pntd.0005005 (PMC5036839; doi:10.1371/journal.pntd.0005005)

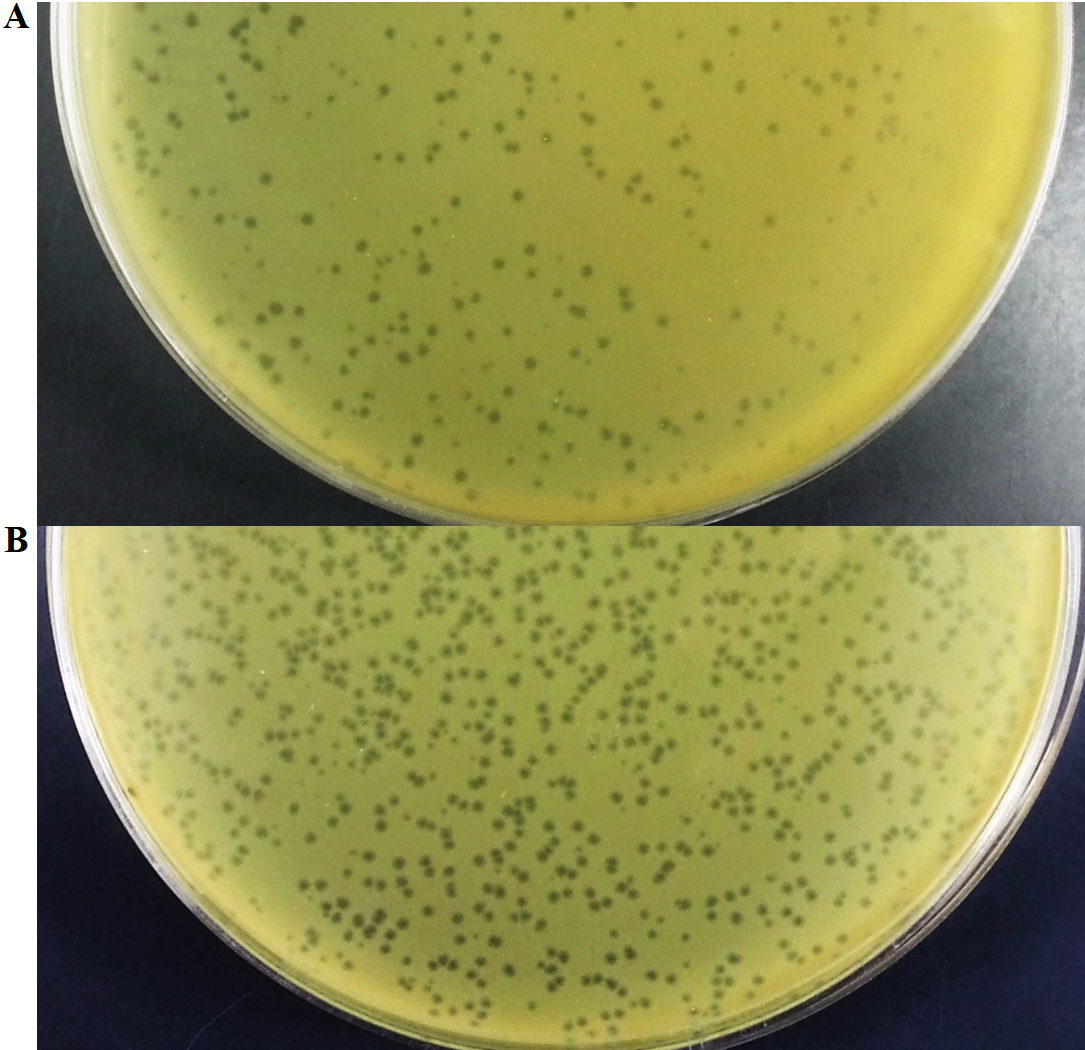

Supplement: S1 Fig — Plaque morphology of soil-isolated phage ΦBp-AMP1 (A) and MMC-induced phage ΦBp-RE1 (B) on the B. pseudomallei 1106a lawns. (TIF) [file pntd.0005005.s001.tif]

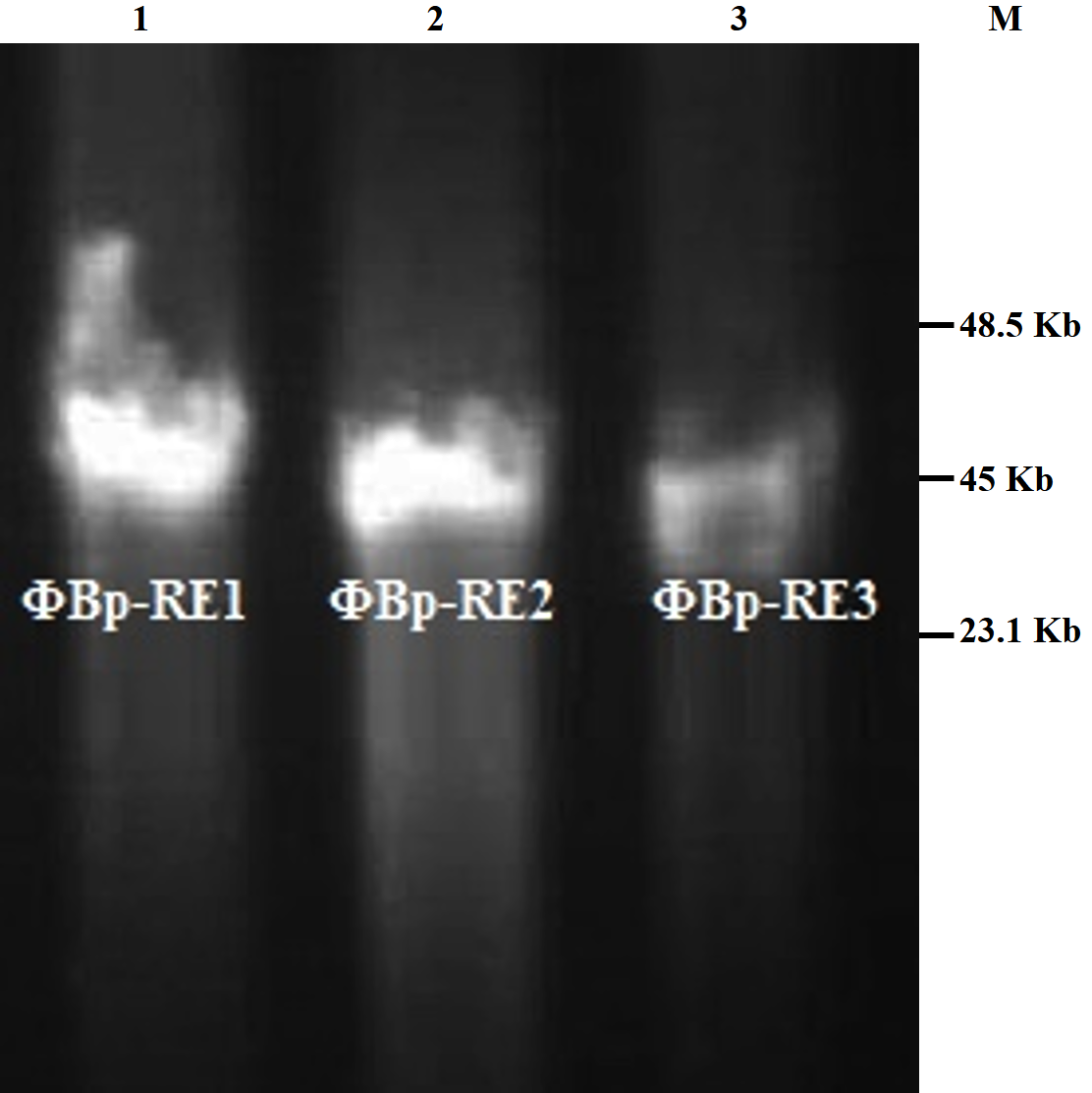

Supplement: S2 Fig — Genomic DNA extracted from MMC-induced phages ΦBp-RE1 (lane 1), ΦBp-RE2 (lane 2) and ΦBp-RE3 (lane 3) were subjected to pulsed-field gel electrophoresis. The genome sizes of these phages were estimated at approximately 45 Kb. (TIF) [file pntd.0005005.s002.tif]
